# The Transactions of the American Medical Association

**Published:** 1861-07

**Authors:** 


ART. III.—*The Transactions of the American Medical Association.* Instituted 1847. Vol. xiii. Philadelphia, 1860. 8vo., pp. 930.

THE annual volume of Transactions of the American Medical Association has an imposing character from its size alone. Thirteen years have elapsed since the first volume of these Transactions was published. They form now a valuable series, comprising much matter of a very superior character, relating to nearly every department of the medical sciences, and a number of ably drawn up reports on the subjects of medical education, and American medical literature, etc. It is true there are to be met with, amid the better portion of their contents, some crude essays which have little, if any positive value, while others also occur of a higher character, but destitute of that originality and research which we had a right to expect in the published transactions of an association which claims to include among its members some of the ablest minds among the medical men of the country. As a whole, however, these Transactions reflect no discredit upon the American medical profession. To judge them fairly, the many disadvantages must be taken into consideration under which the physicians of this country have heretofore labored, and the very inadequate field and opportunities that they have been able to command for close and extended observation in the directions best adapted to enlarge the boundaries of scientific and practical medicine.

Whatever, however, may be the judgment passed upon the thirteen volumes of Transactions that have been issued by the Association, viewed as a collection of papers, pathological, therapeutical, chirurgical, physiological, and hygienic, or as embracing a series of critical and educational reports—whether that judgment be favorable or otherwise—it must be recollected that they afford a very inadequate criterion of the actual workings of the Association, and of the amount of success which has attended its efforts for the attainment of the more important of the objects for which it was organized. They tell little or nothing of what it has done to elevate the character and standing of the American medical profession, for the enlargement and extension of medical education in our midst, and for the promotion of an original national medical literature. To determine how far it has been instrumental in the promotion of all or either of these objects, the condition of the profession at the present time must be contrasted with its condition when the Association came into existence. The increase that has taken place of late years in the facilities for medical instruction must be taken into consideration, together with the zeal exhibited by the student of to-day to avail himself of the new sources for the acquisition of professional knowledge placed within his reach. The few original works of any value in either of the departments of medicine, surgery, or obstetrics, by American authors, that were in existence even as late as 1848, must be compared with the many, and most of them of a very high character, which have been issued from the press within that period. Such a comparison will show that a manifest improvement has been gradually taking place for some years past in everything relating to the character, education, and skill of the members of the medical profession throughout the United States, and a very decided increase in the number and value of our native medical publications. How far this is fairly attributable to the agency of the American Medical Association may perhaps be questioned. Some may even be found who will insist that precisely the same thing would have occurred even had the Association never been called into existence, as a necessary consequence of the more general diffusion of education, the improvements that have taken place in the means and processes of instruction, and the greater activity of the human intellect in the pursuit of every species of knowledge, which is exhibited on all hands. Still, we think there can scarcely be a doubt that a powerful and most beneficial agency has been exercised by the Association in bringing about whatever of advance may be discovered to have taken place in medical education and literature in the country.

The thirteenth session of the Association, the proceedings of which are chronicled in the volume before us, was opened by a very sensible discourse from the retiring president, Dr. Henry Miller, of Kentucky.

After a notice of what had been done toward carrying out the instructions of the Association in regard to obtaining legislative enactments for the more effectual suppression of the crime of abortion, the discourse proceeds to a brief consideration of the history of medical education in the United States, of the broad line of distinction between the manner in which the education of the physician is conducted here and in Europe, the efforts that have been made for its improvement by the Association, and the impediments which have existed and still continue to exist to the carrying of the improvements proposed into successful operation. The views advanced by Dr. Miller are in the main sound, and his suggestions pertinent and judicious.

The first department of the volume before us—after the journal of proceedings—is that occupied by the section of Medical Topography, Epidemic Diseases, etc. It comprises two reports: the one on the Medical Topography and Epidemics of the State of New York, by Dr. Joseph B. Smith; the other on the Medical Topography and Epidemics of North Carolina, by Dr. James Dickson.

Both these reports are ably drawn up, and the contributions they present to the general fund of medical knowledge are highly respectable, and at the same time well digested and systematically arranged. The facts embodied in reports like these, collected by different observers, from different sources, in various localities, and among, to a very great extent, dissimilar classes of people, are well adapted to increase our acquaintance with the history—pathological and therapeutical—of the same class of diseases, according as they occur endemically, amid the different climatic, meteorological, local, domestic, and social conditions in which we find different communities to be placed; and the modifications, impressed through the agency of these different circumstances upon their character, march, and termination, enriching thus and in various other ways our acquaintance with general pathology and with prophylactic medicine.

The report of Dr. Smith skims over a very wide field of inquiry in respect more especially to the first department of its subject, presenting a view of the objects and purposes of medical topography, of the geography and hydrography of New York, and of the geological formations of the State, with the literature of the subject. The report contains a notice of the varieties and distribution of the ores, soils, and mineral springs of New York; of its floral districts and fauna, with the literary history of each subject; an account of the meteorology of the State, in respect to its several physical regions, illustrated by copious statistical tables. It presents, also, a brief notice of the ethnography of the State, embracing a sketch of the discovery of the Hudson River and territory; epochs of the history of the latter; character of its aborigines; its Dutch and Anglo-Saxon population; effects of the intermarriages of European immigrants into the United States since the Revolution; national character of the American people, etc. etc. The brief sketch of these several subjects, compressed as it is within the narrow compass of eighty-eight pages, is by no means devoid of interest and instruction. The whole of this department of the report is indeed drawn up with much skill and ability, and forms a very appropriate and useful introduction to the history of the epidemic diseases of New York, which forms the second department of Dr. Smith's report.

The epidemic diseases of the State are arranged by the doctor in *three* classes: 1st, contagious; 2d, infectious; and 3d, meteoratious. This arrangement is attended with the disadvantage of not being based solely upon either the causes or the characteristics of the diseases embraced in it, but partly upon both these circumstances. Small-pox, varioloid, varicella, measles, scarlatina, etc., it is very true, may be ranked as diseases which are propagated each by a specific poison emanating from the bodies of those who already labor under it. But it is very certain that all of these diseases occasionally originate also spontaneously; that as epidemics their wide-spread prevalence must be due to the influence of some peculiar meteoration or morbific constitution of the atmosphere; that in their character they are liable to an important modification, while their prevalence at particular places is promoted by circumstances of a strictly local character. Hence, when they occur epidemically, they may be placed in the third class with as much propriety as in the first. As infectious diseases, Dr. Smith includes such as owe their origin to koino-miasma, as the periodical fevers, with the yellow fever, and dysentery; to idio-miasma, as typhus and typhoid fevers, etc.; or to a combination of both miasmata, as the compound fevers. There is a large accumulation of facts which would seem very clearly to show that it is not from a simple malarious cause, whether this have a paludal, special, or domestic source, that we are to seek for the origin of the diseases belonging to Dr. Smith's class of infectious maladies, but that they are due to the presence of the same morbific meteoration to which the maladies included in his third class—influenza, typhoid pneumonia, diphtheria, erysipelas, and Asiatic cholera—are supposed to owe their origin. In no other manner can we satisfactorily account for the occurrence of the so-called infectious diseases, every year, at the same season, in certain neighborhoods or sections of country, for a long series of years, and then suddenly their disappearance, in part or altogether, from these places, while, after a longer or shorter period, they again make their appearance with probably increased severity, and having a wider spread than formerly; and this without any appreciable change in the conditions by which it might be supposed that koino- or idio-miasmata would be generated. The fact is, that a careful examination and accurate collation of a large mass of observations bearing upon the etiology of diseases, as well endemic as epidemic, which have been recently accumulated, would seem very clearly to indicate that, if they do not actually originate in certain thermometrical, hygrometrical, and barometrical conditions of the atmosphere, conjoined with, as an essential morbific element, a certain abnormal but as yet inappreciable change in its constitution, still by these conditions the sphere of their prevalence is increased, and the severity of their types respectively augmented; and to affections which ordinarily occur only sporadically, or as very limited endemics, are imparted an increased malignancy of character, and a strictly epidemic or wide-spread erratic prevalence.

The history given by Dr. Smith of the periodical fevers of New York may be consulted with profit, as may also his account of typhus and typhoid fevers, typhoid pneumonia, diphtheria, and erysipelas.

In the report of Dr. Dickson will be found a very excellent and full account of the bilious remittent fever of North Carolina, of typhoid fever and jaundice, and of pneumonia as it occurs in the latter State in its simple inflammatory and typhoid forms. The report contains also an interesting notice of cerebro-spinal meningitis as it prevailed epidemically in some parts of the State of New York during the years 1856 and 1857. Dr. Summerell, of Salisbury, describes a very unusual form of this affection observed by him. It commenced with nearly all the pathognomonic symptoms of what has been called the catarrhal fever of children, in its more severe form. These symptoms were followed in many cases by an erysipelatous eruption, more or less extensive, on some portion of the face, which eruption was always a favorable indication; while in other cases they were followed by bronchitis or pneumonia, when the disease assumed a more severe, but by no means unfavorable aspect. In other cases, again, the catarrhal symptoms were succeeded by those of cerebro-spinal meningitis, and these cases had in nearly every instance a fatal termination.

The proceedings of the Surgical Section are next in order. In these, three reports occur, namely, on the various Surgical Operations for the Relief of Defective Vision, by Dr. M. A. Pallen, of Missouri; on the Improvements in the Art and Science of Surgery in the last Fifty Years, by Dr. Joseph N. McDowell, of Missouri; and on Morbus Coxarius, or Hip Disease, by Dr. Lewis A. Sayre, of New York.

The first of these reports presents a very fair, if not an exhaustive review of what has been done by the surgeon for the removal of certain defects of vision, being supplementary to the report presented by Dr. Pallen at a former meeting of the Association. The present report is not, however, in our opinion, any more than was its predecessor, such a one as is best adapted to promote the objects of the Association, and give character to its transactions. Not because it is destitute, in a certain sense, of intrinsic value, but from its entire want of originality in any one particular. All that it contains of any importance may be found in standard systematic works on ophthalmic surgery, to which every oculist has or may have ready access. It was not, certainly, for the preparation and publication of mere compilations on any given subject, like that of Dr. Pallen, the Association was organized, but rather for the investigation of the various unsettled questions in respect to the different branches of medical, surgical, and obstetrical science and practice, by new series of observations and cautiously-conducted experiments.

The subject of the second report is one invested with a good deal of interest, and, were it handled with sufficient ability, might be rendered highly instructive. The Association has, certainly, not evinced much judgment in the choice of its expositor, on the present occasion, nor any great discrimination in admitting into the printed Transactions the report he has furnished of his labors. Dr. McDowell can scarcely be said to have presented either a full or faithful exposition of the actual improvements that have been effected in the science and art of surgery, during the last half century, with a notice of their relative importance; much less can he be credited with a fair and descriminative criticism of the several modes of practice, and modifications of apparatus and instruments which, within that period, have been brought forward by surgeons of eminence in Europe and the United States—their value being tested by the result of actual observations, and, so far as they can be obtained, well-authenticated and properly-digested statistics. Not only has Dr. McDowell signally failed in the accomplishment of the leading object of the inquiry submitted to him, but what he has communicated as his report is couched in language characterized by a want of proper dignity, as well as of the clearness and conciseness so essential in the discussion of all subjects of a scientific character.

The third report—on morbus coxarius or hip disease, its nature, causes, symptoms, march, and treatment—is a particularly able one. Its chief interest, independent of the accurate delineation of the disease and its accidents, consists—1st. In the position assumed by Dr. Sayre, that the affection known as morbus coxarius is not always or generally the result of a strumous cachexia as is generally supposed, but that, in the great majority of cases, it is the consequence of accidental causes occurring in patients of a healthy, often robust constitution—the attenuation, anæmia, and vitiated nutrition exhibited by the patients in the progress of the disease being always consecutively developed simply as a result of the latter. 2dly. In the facts and arguments adduced to prove that the true cause of the shortening of the limb, in hip disease, is not an upward dislocation of the head of the femur, but a destruction of the latter, and an enlargement of the acetabulum from caries. 3dly. The suggestions as to the advantages which will result from a removal of the morbid effusion, either by puncture or subcutaneous incision of the articular capsule, by which, during the second stage of the disease, the cavity of the hip-joint is distended, and the irritation of the part enhanced and prolonged. 4thly. In the proposal made to excise the diseased portions of the head of the femur so soon as caries has been positively made out to exist in it. We have to regret that the restricted space allotted for this review will not permit of our attempting an analysis of Dr. Sayre's report.

We come next to the proceedings of the Section of Practical Medicine and Obstetrics, embracing a report on the Influence of Alcoholic Drinks in the Development and Progress of Pulmonary Tuberculosis, by Dr. N. S. Davis, of Illinois; another on the Education of Imbecile and Idiotic Children, by Dr. H. P. Ayres, of Indiana; and a third on Inebriate Asylums, by Dr. C. McDermot, of Ohio.

Of the report of Dr. Davis, we can afford room only for the general deductions which he has drawn from the facts, experiments, and clinical observations that have come under his notice. In support of the correctness, generally speaking, of these deductions, we may remark, a much more extended series of statistics than those referred to in the report could be adduced. The deductions of Dr. Davis are as follows:—

"1st. That the development of tubercular diseases is facilitated by all those agents and influences, whether climatic or hygienic, which directly or indirectly impair or retard the metamorphosis of the organized structures, and the efficiency of the excretory functions.

"2d. That observation and carefully-devised experiments both show that the presence of alcohol in the human subject, notwithstanding its temporary exhilaration of the cerebral functions, positively retards both metamorphosis and elimination.

"3d. That neither the action of alcoholic stimulants on the functions of the human body nor the actual results of experience furnish any evidence that these stimulants are capable of either preventing or retarding the development of tubercular phthisis."

The report on the education of the imbecile and idiotic is one of considerable length. The author of it has collected and arranged with considerable industry the leading facts that have been thus far elicited in respect to the general character of idiocy, its classification and degrees; the number and condition of idiots in the United States; the causes of mental imbecility, and the susceptibility of those afflicted with it in any of its forms to the influence of education; with a general summary of the results that have been already made to elevate these unfortunates to the rank of useful members of society, so far, at least, as can be done by rendering them competent to minister to their own support, and at the same time enhancing, as far as possible, their capacity for and sources of happiness. The report closes with a short notice of cretinism—a subject of the deepest interest to the philanthropist, and one having a very close connection with that of idiocy.

In the presentation of so able a digest of what is at present known in reference to the education of the different classes of idiotic children, Dr. Ayres has performed a timely and acceptable task. The subject is one which is now claiming the attention of philanthropists in all portions of the civilized world. The attempt to carry it out to any permanently useful results can be accomplished only by rendering more exact our acquaintance with the true nature and causes of idiocy in its various grades, and the extent to which those laboring under either of these grades are susceptible of physical, moral, and intellectual improvement by the application of proper educational means. It is the members of the medical profession who are looked to, most confidently, for effective assistance in facilitating the means adapted for the amelioration of the condition of the idiots of our country—a labor which, for its accomplishment, appeals to them as philanthropists, as investigators of the physiology and philosophy of mind, and as the legitimate curators of man's mental as well as of his physical infirmities and diseases.

The report of Dr. McDermot treats of another effort of man for the benefit of his fellow-creatures, having its birth in our own times—times so fruitful in every enterprise which has for its object the relief of human maladies, be they moral or physical. Habitual intemperance being no longer viewed as a persistent, voluntary use to excess of intoxicating drinks—a use which the unfortunate inebriate has it in his power, with but little effort, at any moment to relinquish—but recognized as an actual disease, constitutional and hereditary, a proper direction has been given to the attempts made to solve the important question of the means best adapted for the effectual and permanent cure of the malady. All experience has shown that so long as the patient is allowed unrestrained intercourse with society, and, in this manner, is exposed to the temptations to partake of intoxicating drinks by which he will necessarily be surrounded on all sides and continually, the suppression of his habits of intemperance will be attended with an amount of difficulty which too often can scarcely if at all be overcome. Hence, it becomes evident that for the recovery of inebriates an asylum is as necessary as it is for the restoration of the insane; where they can be guarded from the possibility of indulging the morbid appetite for alcoholic stimulants, while they are, at the same time, subjected to such medicinal and moral treatment as shall be adapted to overcome that appetite, administered under the direction and supervisorship of a proper medical officer. To urge the importance of such an asylum, and briefly sketch the benefits which the asylums for inebriates already in existence have produced, is the subject of the report of Dr. McDermot. It is one which will amply repay the time spent in its perusal.

The remaining reports contained in the present volume of Transactions are one from the standing committee of Medical Education, presented by its chairman, Dr. D. Meredith Reese, of New York; another from the committee on American Medical Literature, by Dr. D. E. Wright, of Tennessee; and a third on American Medical Necrology, by Dr. C. C. Cox, of Maryland.

If it were possible to accomplish the reform in the professional education of those who in this country would assume the office of physician by simply a series of annual reports, it is very certain that the American Medical Association, long before now, would have been able to rest from one, at least, of the important tasks assigned it. All must admit that it has labored well and assiduously to indicate the changes necessary in the schemes, periods, and means of instruction pursued in our medical schools, in order to render such instruction adapted to prepare fully their pupils for the discharge of the duties that will devolve upon them on their entrance into the medical profession. Nor can it be denied that the Association has, with commendable zeal, urged upon the several faculties having the instruction of medical pupils in charge, the adoption of the means alluded to.

That the series of very able reports on medical education presented to the National Association, and published in its Transactions, have been productive of good will not, we believe, be called into question. The force of the facts and arguments presented in them have had, it is very clear, we think, a very decided influence in the improvement of medical education in our midst, but not, unfortunately, to the extent that could be desired.

In the present volume of Transactions, in addition to the report of Dr. Reese, of New York, there is another on the same subject; that, namely, of the committee appointed to confer with the teachers of our medical schools, presented by Dr. Thomas W. Blatchford.

By the first of these reports it is made an indispensable requisite to the reception of any one as a student of medicine, that he shall be at least seventeen years of age, of good moral character, possessed of a good English education, shall be able to read and translate the Latin language, and have an elementary knowledge of Greek. His qualifications to be tested by actual examination. It requires, in all, *four* years of study, including attendance upon lectures: the fourth year to be devoted mainly to clinical hospital instruction, in medicine, surgery, and obstetrics. It requires attendance upon *three* complete courses of lectures in a recognized school. Candidates to be admitted to examination after *three* full years of instruction in all the branches which are required to be taught, excepting clinical medicine. It extends the lecture term through the full period of *nine* months in each year—the term to be divided into *two* parts or sessions, the first being chiefly occupied with instruction in the *elementary* branches; the second, in the practical and more advanced branches. The pupils of the first session to constitute the *junior*, and those of the second session the *senior* class. Not more than *four* lectures to be attended daily by either of these classes; each lecture being preceded by a recapitulary series of examinations upon the subjects of the lecture of the preceding day. The number of teachers in each school to be increased in accordance with the large increase in the number of branches required to be taught. It demands the same examination previously to admission into the senior as into the junior class—the examination for the promotion of a junior to have reference both to the fullness of his preliminary education, and the amount of his improvement in the branches taught in the primary session: admission into the senior class to be predicated upon the satisfactory result of the examination. A similar examination is appointed at the commencement of each session to test the improvement made during the preceding term. The final examination for graduation to be of each candidate separately before the entire faculty, and of commissioners appointed by the medical society of the State in which the school is located—a certificate of approval from the latter being required before the degree can be conferred. No candidate to be admitted to examination before the expiration of the regular lecture term. No school to be recognized which has not access, for the clinical instruction of its pupils, to a hospital containing at least *eighty* beds. The so-called "college clinics" are denounced as inadequate for clinical instruction. The titles of the several chairs in a school to indicate the actual branch or branches taught by each.

The foregoing suggestions are in the main judicious and practicable. The result of their inauguration as the basis of an improved and more extended system of medical education in this country would, we are well persuaded, be in the highest degree beneficial.

The report of the committee of conference lays down, as the foundation of a proper reform in medical education, the following propositions :—

1st. A term of study for each candidate for the doctorate for the full and entire period of *three* years, under the direction of a recognized practitioner, and a *bona fide* attendance during two terms, in different years, upon full courses of lectures delivered in a medical school recognized as one regularly organized by the Association. 2d. A permanent record to be kept, containing the signature of each matriculant, with his age, the time he commenced the study of medicine, and with whom, also the full description of any evidence of previous education he may possess. 3d. The necessity of hospital clinical instruction as an essential part of medical education ; upon this, each candidate for graduation must have attended regularly for at least *four* months. 4th. The attendance upon the examination of all candidates for the doctorate of delegates from the medical society of the State, the favorable vote of whom shall be necessary to the graduation of such candidates. 5th. No medical school to be recognized as regularly organized unless represented at a meeting of the Convention of Medical Teachers, etc., or which does not comply with the regulations and recommendations of the American Medical Association. 6th. Evidence of suitable preliminary education to be positively required from all matriculants into medical schools. A final recommendation of the report is, that every proper effort be employed to call the attention of individuals, as well as legislative bodies, to the importance of endowments, whether of medical colleges or their respective professorships.

The whole of the suggestions and propositions made by the two committees above referred to were, we believe, unanimously adopted by the Association. In their general features the recommendations of both committees correspond; the difference between them is in details only, and these mostly of secondary importance. We shall wait patiently to see how far they will be carried out in good faith by the faculties of our medical colleges, and a compliance with them insisted upon by the profession generally. In respect to the good that would result from their faithful execution upon the part of all concerned there cannot, we think, be the most trifling dispute.

The report of Dr. Wright on Medical Literature is, to say the least of it, a very sensible document. It attempts, and it appears to us with success, to prove that there has arisen of late years an increased demand for original professional works by American writers, and a consequent increase in the supply. During the last two or three years it appears that a very nearly equal number of new American and English works, in different departments of medicine, have made their appearance in the United States; while during the six months immediately preceding the date of the report before us the new works on medicine by American writers outbalance the republication of foreign medical works in number, while they present a character for intrinsic value that will scarcely lose anything when compared with their competitors from abroad. It is shown that in this country the demand is almost exclusively for works in which the facts, observations, and improvements, the raw materials, so to speak, which have been developed and fashioned by the members of the medical profession generally, at home and abroad, are collected and arranged so that their practical application, in the correction of received theories or in modifying the prevailing system of therapeutics, is presented to the reader for immediate use. Strictly original monographs in either of the departments of medical science or practice have but few readers among us, and perhaps as few producers. There are, indeed, in the United States, but a small number of medical men who have either the inclination or the time, even if they have the capacity and the means, to engage in the search after new truths in medicine, or by a long series of careful observations, through fields of sufficiently extended limits, to develop improved and more certain plans for the treatment of any particular malady or of disease generally. Our medical teachers are most usefully occupied in indoctrinating their pupils into the principles of medical science and practice as they now exist: inculcating such of the prevailing doctrines as to them seem most correct, and perfecting the therapeutic directions laid down by the professional authorities of the day, from the teachings of their own experience as private practitioners, or perchance as chief of some large general hospital. Their teaching must be chiefly elementary—theirs is not the task of some distinguished professors abroad, to discuss new views and new developments of scientific questions before an audience of scientific men. Our medical writers, also, when they attempt anything beyond an essay for a professional journal, or a report to their county or State society, restrict their efforts mainly to the preparation of a systematic treatise on medicine, surgery, or obstetrics, more or less comprehensive; in the preparation of which they employ materials furnished, not by their own, but by the labors of others: systematizing and utilitizing professional knowledge already existing in detached parts, for the benefit of the less industrious members of the medical community, as well as of such as have not the leisure to consult, even if they have access to the sources from whence that knowledge is to be derived. Direct practical utility is almost the only criterion of excellence in medical literature in this country, and the professional books which are the most in demand are either those which offer, on the emergency, prompt practical counsel to the physician, or which can be used as text-books in facilitating the acquisition of sufficient knowledge to secure a doctor's diploma. For monographs of original research on abstruse topics which cannot be reduced at once to a positively practical application, the American medical profession supplies few, if any readers, and such will continue to be the case until a body of medical men shall arise in our midst willing to devote their time and talents solely to the cultivation and advancement of medicine and its collateral sciences, as important branches of liberal science, equally interesting from simply the truths they embrace, as for the benefits they are adapted to confer upon the human family.

The report on Medical Necrology presents brief notices of some of the more prominent physicians who have died within the last few years. We are thankful for the meagre details furnished by Dr. Cox, while we could have desired more full notices of the lives and labors of more than one of the recently departed, who by their moral worth adorned their profession, and by their labor extended materially its bounds.

